# The devil is in the details: trends in avoidable hospitalization rates by geography in British Columbia, 1990–2000

**DOI:** 10.1186/1472-6963-6-104

**Published:** 2006-08-16

**Authors:** Denise Cloutier-Fisher, Margaret J Penning, Chi Zheng, Eric-Bené F Druyts

**Affiliations:** 1 Centre on Aging, University of Victoria, Victoria, BC, Canada; 2 Department of Geography, University of Victoria, Victoria, BC, Canada; 3 Department of Sociology, University of Victoria, Victoria, BC, Canada; 4 Western Regional Training Centre for Health Services Research (WRTC), Department of Health Care and Epidemiology, University of British Columbia, Vancouver, BC, Canada

## Abstract

**Background:**

Researchers and policy makers have focussed on the development of indicators to help monitor the success of regionalization, primary care reform and other health sector restructuring initiatives. Certain indicators are useful in examining issues of equity in service provision, especially among older populations, regardless of where they live. AHRs are used as an indicator of primary care system efficiency and thus reveal information about access to general practitioners. The purpose of this paper is to examine trends in avoidable hospitalization rates (AHRs) during a period of time characterized by several waves of health sector restructuring and regionalization in British Columbia. AHRs are examined in relation to non-avoidable and total hospitalization rates as well as by urban and rural geography across the province.

**Methods:**

Analyses draw on linked administrative health data from the province of British Columbia for 1990 through 2000 for the population aged 50 and over. Joinpoint regression analyses and t-tests are used to detect and describe trends in the data.

**Results:**

Generally speaking, non-avoidable hospitalizations constitute the vast majority of hospitalizations in a given year (i.e. around 95%) with AHRs constituting the remaining 5% of hospitalizations. Comparing rural areas and urban areas reveals that standardized rates of avoidable, non-avoidable and total hospitalizations are consistently higher in rural areas. Joinpoint regression results show significantly decreasing trends overall; lines are parallel in the case of avoidable hospitalizations, and lines are diverging for non-avoidable and total hospitalizations, with the gap between rural and urban areas being wider at the end of the time interval than at the beginning.

**Conclusion:**

These data suggest that access to effective primary care in rural communities remains problematic in BC given that rural areas did not make any gains in AHRs relative to urban areas under recent health sector restructuring initiatives. It remains important to continue to monitor the discrepancy between them as a reflection of inequity in service provision. In addition, it is important to consider alternative explanations for the observed trends paying particular attention to the needs of rural and urban populations and the factors influencing local service provision.

## Background

Equity is defined as the fair and just distribution of resources. In Canada this means equal access (or equal service) for equal need [1]. Health care systems that limit access or fail to provide equitable access to care for populations have been said to accentuate social disparities in health [1]. Arguably, the real test of equity of access involves determining whether there are systematic differences in use and outcomes among various groups in society, and whether these differences result from financial or other barriers [2]. Making progress towards greater equity in service provision requires understanding how well the health system is able to distribute services to populations and sub-populations on the basis of need. The 1984 Canada Health Act guarantees reasonable access to medically necessary care (provided by physicians and hospitals) to all Canadians. While this Act minimizes barriers to access that are financial in nature, geographic and other barriers (e.g., cultural) remain largely unaddressed.

Research reveals that geographic barriers represent a major source of inequitable access to care [3,4], with health service utilization being inversely related to physical access (i.e., travel distance to obtain care) [4]. It is also reasonably well accepted that the range and type of care provided varies by geography. Larger communities provide a broader range of services, while smaller urban areas and rural communities offer fewer service alternatives and/or reduced access to care. Some of the most often noted health service system deficiencies cited in smaller communities include the following: lower ratios of general practitioners and specialists per capita, fewer hospitals (and beds), reduced availability of allied health professionals (such as home support workers, therapists and counsellors), and limited respite care, palliative care and mental health care options [5,6]. Human resource retention problems and practitioner isolation are also frequently noted [7].

From the inception of universal health insurance in Canada, provincial and territorial governments have been interested in enhancing equity in service provision, as well as citizen participation, accountability of decision-makers, service efficiency and cost-savings [8,9]. These goals remain prominent. Regionalization, or the restructuring of decision-making authority and responsibility for health care delivery within local communities has been one of the primary mechanisms adopted to address barriers limiting equitable access to care during the past decade or more. In the province of British Columbia (BC), for example, the Royal (Seaton) Commission on Health Care and Costs 'Closer to Home' Report [10] and the 'New Directions for a Healthy BC' initiative [11] asserted the need for regionalization to address several goals including the integration and coordination of health services at the regional and community scale, devolved decision making and control to local communities and citizen empowerment [12].

In 1997, a profound set of changes altered the way that health care was organized and delivered in BC: the Health Authorities Act established 52 local health authorities made up of 11 Regional Health Boards (RHBs), 34 Community Health Councils (CHCs) and 7 Community Health Service Societies (CHSS) to look after health planning and service delivery across the province [13]. RHBs were given responsibility for metropolitan or urban service provision, while CHCs and CHSSs were jointly responsible for planning and service delivery in rural and remote areas. In 2001, following a change in government, a new round of restructuring resulted in amalgamation of the province's health authorities into five larger regional health authorities and fifteen health service delivery areas, thereby re-centralizing power and authority with the provincial government and reducing local decision making [12].

Primary care is considered to be one pathway through which inequalities (geographic, economic, social) influence population health [14]. Consequently, governments at all levels have identified primary care reform as a top priority to move the health system away from a sickness model and towards a wellness model supported within a population health framework [15,16]. By definition, primary care is the first level, or the most common point of contact that individuals have with the health care system. The chief focus of primary care is the identification, diagnosis, treatment and management of health concerns [16].

Historically, primary care has been considered the domain of family physicians or general practitioners, working in group or solo practices, and acting as gatekeepers to the health care system. However, primary care is increasingly viewed more holistically, as care provided by nurses, social workers, therapists, family physicians and others in community health centre settings [16]. Due to the historical development of primary care, reform efforts tend to focus on increasing access to care, incorporating multidisciplinary teams, improving information and technology systems, shifting physician remuneration from fee-for-service to alternative payment methods (e.g., salary or capitation), and enhancing coordination and integration with other health services (i.e., in institutions and in communities) [16,17]. The integration of services, especially within primary care, is a keystone to health care reform. Research from Quebec emphasizes the importance of regionalization to provide the structure and leadership required for successful primary care reform initiatives [18].

To assess the adequacy, efficiency and quality of primary care within the broader health system, one indicator that researchers have focused on is 'avoidable' or 'preventable' hospitalizations [19-25]. With some qualifications, avoidable hospitalizations are said to represent a range of conditions for which hospitalization should be avoidable, provided that individuals have access to timely and effective primary care. More specifically, they represent a group of hospital episodes that could be treated in a primary care setting (i.e., a physician's office or community health clinic), provided that individuals are able to access these facilities at the appropriate time and the appropriate care is prescribed [26]. Consequently, in any given geographic area, the expectation is that avoidable hospitalizations should be lower when people are receiving the primary care they require. Conversely, in areas where access to medical care is more limited, rates of avoidable hospitalizations tend to be higher [20].

Research findings have also confirmed that poor primary care, reduced access to care and diminished resources act together to increase avoidable or preventable hospitalizations [27]. AHRs have been found to be inversely related to the supply of primary care physicians in both core metropolitan and rural communities [28]. For example, Parchman and Culler report finding higher rates among persons living in US counties considered to have a shortage of primary care [29], while Lin et al. report higher AHRs in rural and remote areas compared with urban areas of BC [4]. Regionalization and other health sector reforms appear to have had an impact on hospitalization rates in Canada in recent years. Hospitalization rates have declined, likely in conjunction with hospital and hospital bed closures implemented over the past three decades [30,31]. From 1991/92 to 1996/97, the number of staffed beds in BC declined by 30%, the number of acute days per 1000 population declined by 28.8%, and the average length of stay declined by 12.9% [31]. Some evidence suggests that AHRs also declined [4,32,33]. Yet, evidence about the extent to which these declines reflect the effects of regionalization and primary care reform and associated reductions in rural-urban inequities in care remains elusive.

While AHRs are an indicator of health system efficiency, it is also useful to consider how local populations (i.e., rural and urban) differ and to consider how they are being impacted by regionalization processes. Not surprisingly, health service system disadvantages that are present in small communities are exacerbated by population trends like demographic aging. For example, many small rural communities exhibit populations with high proportions of older adults that may be two to three times higher than provincial and national averages [34]. Some studies suggest that rural residents experience a higher prevalence of chronic conditions and higher premature mortality rates compared to their urban counterparts, and also have higher death rates from unintentional injuries, chronic obstructive pulmonary disease, and suicide [35,36]. It is also evident that the most rural and the most urban areas are of greatest concern in terms of the health status of local populations [35]. Mainous and Kohrs studied rural and urban adults in Kentucky and found that rural dwellers age 65 and over had poorer health status, poorer physical and social functioning, and reduced mental health, although there were fewer significant differences when comparing the overall populations between rural and urban areas [36]. A study comparing rural and urban older adults in Manitoba found no significant differences in self-rated health, but noted that rural elderly individuals were more likely to be satisfied with their health [37].

It may be more important to consider specific rural service contexts vis-à-vis the characteristics of particular rural populations (i.e., specific towns, cities and villages with higher proportions of seniors in their populations). The inconclusive evidence about whether rural populations are more or less healthy than their urban counterparts is partially explained by variations in regard to how 'rural' is defined, and given that the variables used to measure 'health' also vary. However, it may be sufficient to suggest that older persons living in rural areas are more vulnerable to regionalization given their situation of 'double-jeopardy,' that is, living in environments with reduced services at a time in their lives where they may have a greater need for care in relation to their age and health status [34]. Benoit et al. conclude that access to rural maternity care was likely made worse by regionalization despite the fact that concern about the scarcity of providers in rural and remote communities predated regionalization [12]. Generally speaking, the effectiveness of regionalization strategies in addressing geographic and other inequities in access to health care remains ambiguous and inconclusive [13].

This research examines trends in avoidable hospitalization rates as an indicator of primary care system access among adults aged 50 and over, living in rural and urban communities in British Columbia during a period of extensive regionalization. Two research questions are addressed: (1) What has happened to avoidable hospitalization rates, relative to non-avoidable hospitalization rates and total hospitalization rates, over time and during a period of health care restructuring and primary care reform initiatives in British Columbia, Canada?; and (2) To what extent do trends in avoidable hospitalization rates differ across rural and urban areas of the province? Specifically, has equity between rural and urban areas improved over time?

If there were no differences in equity of service provision, trends with regard to AHRs in rural and urban areas would be expected to overlap. On the basis of the literature and known trends in the availability of primary care, it is anticipated that AHRs would be higher in rural areas than in urban areas prior to reform. To the extent that regionalization efforts and primary care reform have been successful, one would expect to see this reflected in reduced AHRs over time. Additionally, if primary care reforms have been effective in reducing geographic inequities in access, one would expect to see decreasing AHRs, as well as greater reductions in AHRs in rural areas compared with urban areas over time.

## Methods

### Data sources

Our analyses draw on administrative health and population data for those aged 50 and over in British Columbia. Administrative health data (including hospital utilization records with geographically referenced data) were accessed through the British Columbia Linked Health Data (BCLHD) resource, a data repository available through the provincial Ministry of Health and distributed to researchers by the Centre for Health Services and Policy Research (CHSPR) at the University of British Columbia. The reliability and validity of hospital administrative records for research purposes are well documented [38-41]. Hospital separation records are generated each time an individual is discharged from hospital, whether treated as an inpatient or outpatient. Data include basic demographic information on clients, assessment records, approved care, direct care records, and billing information. Geo-code data include information on individual area of residence, including local health area (LHA); this allows merging of these data with the hospital separation records. Files are linked using person-specific unique identifier codes. With due consideration to exclusions and recording error, these data can be considered to represent the entire population rather than a particular sample (since all events of hospitalization for this age group are captured). Under these terms, statistics are used to help identify noteworthy areas of difference. Population denominator data were derived from BC Statistics [42]. As population estimates are updated continually, 11 years of annual population data were generated from the most current Population Estimates (1986–2003) and Projections (2004–2031).

### Sample

Hospitalization data were drawn from the population of health service users (derived from a registry of users of all publicly funded health services) aged 50 years and older who lived in the province each year from April 1, 1990 through March 31, 2000. Since all BC residents are covered by the province's publicly insured medical and hospital service plan and the vast majority of residents draw on health services of one type or another each year, these data effectively represent the entire population of the province (which ranged from 3.29 million people in 1990 to approximately 4.0 million people in 2000) [43]. Individuals whose area of residence was unknown or missing were excluded. The number of individual records that were deleted for this reason ranged from a low of 2,312 in 1998 (0.75% of total records) to a high of 7,935 in 1990 (2.74% of total records). The trend toward decreasing missing data over time might reflect increasing awareness of the importance of these data and the need for the most complete hospitalization records possible for research and other purposes.

### Measures

Our analyses assess population-based changes in AHRs over time. Avoidable hospitalizations were defined based on work conducted by Weissman et al. who used multiple selection criteria to identify twelve conditions for which hospitalization was considered avoidable given effective primary care (e.g., ruptured appendix, asthma, congestive heart failure, malignant hypertension, cellulitis, diabetes – see Table 1) [23]. Timely and appropriate ambulatory care is viewed as being key to preventing illnesses, controlling acute episodes or managing chronic illnesses to prevent them from deteriorating into the need for hospitalization. Older populations have disproportionately more avoidable hospitalizations than younger cohorts [22,27], providing a rationale for examining trends among older adults, although AHRs were originally developed to describe hospitalizations among the population aged 0–64.

The primary diagnosis associated with the first avoidable hospitalization separation in each fiscal year for each individual patient was used to define a single 'avoidable hospitalization' claim. Thus, the numerator referred to whether or not a given individual's first hospitalization during the year was classified as avoidable. This reflects a more conservative estimate of AHRs than actually occur, but avoids counting multiple hospitalizations for the same avoidable condition for one person in a given geographic area. The original ICD-9 codes used by Weissman et al. [23] were updated based on the 10^th ^revision, ICD-9-CM. Age-sex standardized utilization rates were then calculated using the University of Manitoba SAS rates macro [44]. All rates were standardized to the 1991 Canadian population. For each of the eleven years, three annual AHRs were produced (rural, urban, and total population aged 50 and over in BC). To provide a context for interpreting AHRs relative to overall trends in hospitalization over the time interval, non-avoidable hospitalization rates (non-AHRs) and total hospitalization rates (Tot-HRs) were also generated.

Rural and urban areas were differentiated based on geographically-based administrative health units within the province of BC. Regional Health Boards (RHB), Community Health Service Societies (CHSS), and Community Health Council (CHC) boundaries were distinct geographic administrative units used for health care planning and service delivery purposes under the health care reforms implemented in British Columbia during the 1990s. As noted, 11 RHBs represent densely populated 'urban' areas of the province, while seven CHSSs and 34 CHCs define less densely populated 'rural' areas of the province. By examining the data, approximately 85% of the province's population reside in areas considered urban and governed by RHBs, while 15% reside in areas considered rural and governed by CHSSs. The boundaries of each are updated from time to time: the geo-references used for defining RHBs and CHSSs in this paper are from administrative decisions as of June 2004. We used these administrative units to dichotomize the hospital separations into rural and urban components.

### Analysis procedures

Joinpoint regression analyses (version 2.7) [45] are used to assess changes in age-adjusted AHRs over time. This procedure, also known as piecewise or segmented regression, fits a model based on the minimum number of joinpoints (points of change in slope) that are observable across a series of rates over time [45,46]. In this analysis, the unit of analysis is the year, with eleven data points being represented (i.e., 1990–2000). Joinpoint regression analyses assume a log-linear model. As Kim et al. [46:350] point out, "(w)hile other approaches could be considered, joinpoint regression is a useful way to summarize trends ... (C)onnecting linear line segments on a log scale allows us to characterize the trends succinctly...(and) also allows us to test for recent changes in trend."

In these analyses we assess whether trends in AHRs are best modelled using a straight line (0 points of change, JP = 0) or whether one (JP = 1) or two (JP = 2) points of change are more appropriate [46]. The program tests the null hypothesis first (0 versus 2 joinpoints); if Permutation Test (PT) results point to rejection of the null hypothesis (joinpoints = 0), then a test for 1 versus 2 joinpoints is applied. If initial results indicate acceptance of the null hypothesis, then a test for 0 versus 1 joinpoint is administered. The decision to restrict the maximum number of change points for the regression line to two was based on the fact that we had eleven years of utilization data. T-tests are used to compare slopes across categories (i.e., rural, urban, total). T-tests were also undertaken to test if AHRs and non-AHRs were significantly different from each other.

## Results

In general non-avoidable conditions account for the vast majority of hospitalizations in a given year (e.g., 94.8% in 1990 and 95.3% in 2000) with avoidable hospitalizations making up the difference (e.g., 5.2% in 1990 and 4.7% in 2000). In 1990, the total number of first hospitalizations among those age 50 and older in BC stood at 173,405 indicating that 20.0% of the population in this age group experienced at least one hospitalization during that fiscal year. The age-standardized rate of claims was 197.0 per 1000 population. By 2000, the total number of first hospitalization claims for those in the 50+ age group was 204,919 with a slightly lower proportion (17.4%) of the population in this age group being hospitalized during that year. By 2000, the age-standardized rate for total hospitalizations had declined to 172.6 per 1000 population.

The first research question that this study addresses concerns what happened to AHRs relative to non-AHRs and to Tot-HRs over time and during a period of health care restructuring and primary care reform in BC, Canada. The number of unique first claims for avoidable hospitalizations ranged from 9,092 in 1990 to 9,642 in 2000. AHRs declined over the eleven-year interval from 10.2 per 1000 population in 1990 to 7.8 per 1000 in 2000 (Table 2). In rural areas, AHRs ranged from 14.4 per 1000 in 1990 to 10.7 per 1000 in 2000 while for urban areas, the corresponding AHRs were 9.7 per 1000 in 1990 and 7.4 per 1000 in 2000 (Table 2).

**Figure 1:**
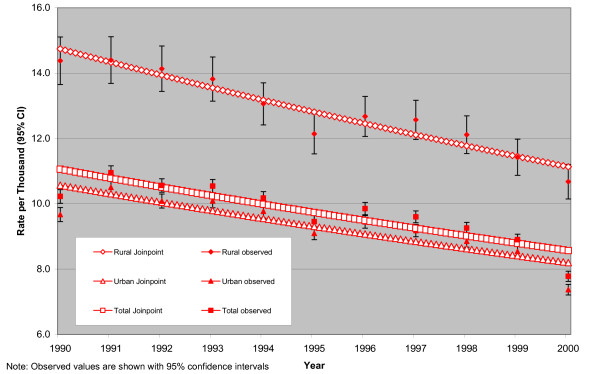
Joinpoint Regression Best-Fit Lines for Avoidable Hospitalization Rates (by Geography, 1990–2000)

Figure 1 and Table 3 depict the joinpoint regression results for AHRs over the study interval. These data show that from 1990 to 2000, AHRs declined significantly and more sharply than total hospitalizations (as revealed by the slope coefficients), with no significant changes in trend (i.e., JP = 0) evident during this period.

Our second research question was concerned with the extent to which trends in AHRs differed across rural and urban areas of the province. For comparison purposes, relative rates were generated (i.e., by dividing rural rates by urban rates for each year). These trends reveal that standardized rates for avoidable, non-avoidable and total hospitalizations are consistently higher in rural areas compared with urban areas over the time interval studied. Furthermore, for avoidable hospitalizations, relative rates for AHRs are 1.3–1.5 times higher for rural areas, while the relative rates for non-avoidable and total hospitalizations are approximately 1.2 times higher for rural areas compared with urban areas. Thus, rural AHRs are consistently higher than urban AHRs and the discrepancy is larger than for non-AHRs and total AHRs.

Examining the relative rates for avoidable hospitalizations over time reveals a fluctuating, parabolic trend. In 1990, the relative rate is 1.49 per 1000 and by 2000 the relative rate is 1.45 per 1000, however; in the middle of the time interval, relative rates in avoidable hospitalizations bottom out around 1.34–1.37 per 1000.

In addition to the comparison of numerical and relative rates, Tables 3 and 4 report the joinpoint regression and the t-test results to assess the statistical significance of differences in slope over the time interval. The joinpoint regression results for AHRs by rural and urban geography reveal a significantly decreasing trend with no joinpoints and where the slope of the line for rural areas is slightly greater than the slope for urban areas (slope rural = -0.028, p = 0.000; slope urban = -0.025, p = 0.001).

A total of six comparisons were made to assess changes in slope (Table 4). In the first three, rural to urban comparisons were made among AHRs, non-AHRs and Tot-HRs. In the second set, slope comparisons were undertaken by geography. For example, AHRs were compared to non-AHRs within the urban category, the rural category and for both areas combined. Comparing the slopes for AHRs between rural and urban areas revealed no significant difference (p > 0.05). Thus, while a comparison of numerical rates and relative rates over time suggest a substantial difference in rural rates relative to urban rates, the t-test results support the conclusion of parallel lines rather than any significant convergence.

**Figure 2:**
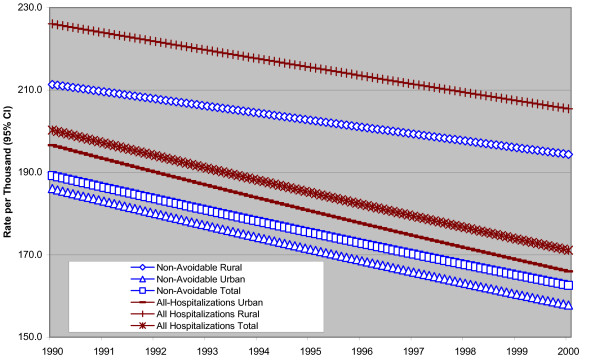
Joinpoint Regression Best-Fit Lines for Non-avoidable Hospitalization and Total Hospitalization Rates (by Geography, 1990–2000)

In contrast, comparing the slopes for non-AHRs between rural and urban areas reveals a highly significant difference (p < 0.005) with a diverging trend by the end of the time interval. In other words, from Figure 2 and Table 4, the joinpoint regression lines are wider at the end of the time period than at the beginning (i.e., diverging) and the difference in slopes is significant between rural and urban areas. A similar result is observed when comparing Tot-HRs for urban and rural areas in BC. In this latter case, the slopes were also significantly different from each other (p < 0.01). Thus, both sets of rates demonstrate decreasing trends with no joinpoints that are diverging over time. In summary, for non-AHRs and Tot-HRs, the declines are not occurring at the same rate for rural and urban areas, with rates in rural areas declining more slowly than rates in urban areas over the time period.

From the second set of comparisons, slopes for AHRs and non-AHRs in urban areas are significantly different (p < 0.01). Comparing AHRs and non-AHRs in rural areas revealed the same result, i.e., a significant difference in slopes (p < 0.01). In contrast, when comparing total AHRs to total non-AHRs the difference in slopes was not significant (p > 0.05). Thus, there are both significant and unique differences in the trends in slopes when comparing AHRs and non-AHRs over the time interval.

According to the joinpoint regression results (Figure 2 and Table 3), for total hospitalizations, the trend is towards significantly decreasing rates with no significant joinpoints throughout the time interval (JP = 0, slope = -0.016, p = 0.000). The total non-avoidable hospitalizations depict the same trend, showing statistically significant declines, with no significant joinpoints throughout the time interval (JP = 0, slope = -0.015, p = 0.000).

In 1990, the non-AHR was 186.8 per 1000 population and by 2000, the rate stood at 164.8 per 1000 population. Figure 2 depicts the trends in Tot-HRs and non-AHRs among those aged 50 and over in BC from 1990 through 2000. The relative rate for non-avoidable hospitalizations between rural and urban areas is 1.16 per 1000 in 1990 and 1.24 per 1000 in 2000. For Tot-HRs, the trends are very similar with the relative rates between rural and urban areas being 1.17 per 1000 in 1990, increasing to 1.25 per 1000 by the year 2000.

To summarize, examining the data by urban and rural geography reveals that despite the apparent convergence in relative rates for avoidable hospitalizations between rural and urban areas over the time period the slopes are not statistically significant different. However, for total and non-avoidable hospitalizations there are statistically significant and diverging trends over the time interval with the gap between rural and urban areas being wider at the end of the time interval than at the beginning.

## Discussion

This study examined trends in hospitalization rates over a period of time (1990–2000) characterized by multiple regionalization initiatives aimed at improving equity and efficiency in service delivery as well as enhancing service coordination. AHRs were selected as an indicator of primary care system access. The results revealed that age-adjusted avoidable hospitalization rates (AHRs) declined in both rural and urban communities across the province in the 1990s. Such declines would suggest positive gains in health system performance and point to the increasing effectiveness of primary care, but such an assertion bears further scrutiny.

The first research question examined AHRs in relation to non-AHRs and Tot-HRs in BC during the 1990s. The findings revealed statistically significant declines in all hospitalizations (both avoidable and non-avoidable) over the time period. Given that it is extremely difficult to establish a causal link between regionalization and declining hospitalizations, alternative explanations for decreases in hospitalization rates over time should also be considered. Other reasons for decreasing rates may be linked to subtle shifts in the historical reliance upon physicians and hospitals at the 'heart' of primary care, toward a broader notion of 'primary health care' that includes other community-based practitioners and even informal sources of care. For example, many communities are beginning to explore the potential for nurse practitioners to become partners in care.

In terms of efficiency, declining hospitalization rates across the board (i.e., AHRs, non-AHRs and Tot-HRs) also suggest the possibility that costs are being reduced within the acute care sector. On the system side, it may well be that any savings due to declining hospitalizations and shorter lengths of stay are a product of reduced capacity (i.e., fewer beds). On balance, it is also possible that any savings that arose were absorbed by increases in related sectors (e.g., physician remuneration or by the application of more intensive treatments or expensive technologies for those who are hospitalized). However, it is unclear whether such changes in composition and form would be sufficient in magnitude to account for the changes in utilization observed over the time period studied. On the population side, it would also be instructive to examine trends in service use among older populations, considering the broadest possible spectrum of service alternatives and complementary data (e.g., from mental health, residential long-term care and related sectors) as well as taking the care provided in the informal sector into account at the same time.

The second research question addressed trends in AHRs by geography (i.e., across urban and rural areas of the province). The findings revealed that AHRs are consistently higher in rural areas than in urban areas. Vertical equity is suggested by the fact that rural areas have higher hospitalization rates for both AHRs and non-AHRs. These higher rates reveal that rural residents are accessing more acute care services. From a health system perspective, higher rates of hospitalization for rural areas are also suggested by policy and planning documents that allocate more hospital days per capita for rural populations in BC in recognition of lower physician/population ratios, fewer hospitals and hospital beds and a smaller range of community-based care options in smaller community settings [47].

However, it is also possible that rural hospitals may inadvertently be promoting higher AHRs as a means of ensuring their viability. To explore this issue more fully, it would be useful to apply a different methodology that takes all hospitalizations for individuals into account (rather than first hospitalizations as employed here), and then compares them by rural and urban areas.

Over the decade, the decline in AHRs may be interpreted in a positive light as suggesting that access to effective primary care is improving throughout BC. Comparing AHRs in rural and urban areas allows for consideration of issues around horizontal equity. Such issues loom large in rural communities, as horizontal equity presumes that there should be no difference in health service provision where health needs are equal. A lack of conclusive evidence about the needs of rural and urban populations (i.e., how needs differ and whose needs are greater) makes it difficult to explain this observation in definitive terms, but calls attention to the importance of examining the specific needs of older rural and urban adults within specific local community service contexts. If the needs of older populations are presumed to be similar regardless of geography, and if resources are equitably distributed, the expectation would be that AHRs would be similar and trend lines would overlap. Overlapping trend lines would suggest access to effective primary care is similar in both geographic contexts.

Evidence that AHRs are higher in rural areas combined with the lack of a significant difference in the slopes of the lines between rural and urban areas, means that inequities in service provision that existed in BC at the beginning of the 1990s, are essentially being preserved over time. The conclusion therefore is that in the absence of considerations of service needs, older rural populations have reduced access to effective primary care when compared with their urban counterparts (as represented by these higher AHRs), and this was no less true at the end of the decade than it was at the beginning.

If the needs of rural populations are greater than the needs of urban populations, then higher AHRs continue to reflect poorer access to effective primary care for residents of rural communities across BC. Thus, the magnitude of the discrepancy between rural and urban areas reflects an equity gap. Even so, a more important question concerns the points at which the discrepancy poses a serious threat in terms of heightening the social disparities between rural and urban populations.

Reflecting for a moment on populations rather than health care systems, it is possible that declining rates of hospital utilization over time might reflect increases in the health of the population (thus, declining need) or changes in health-seeking behaviours, rather than changes in health care policy or associated changes in service delivery (e.g., access, availability and eligibility criteria) related to regionalization. There are substantial debates in the literature today about whether older adults will be more or less healthy in the future compared with current cohorts. Not surprisingly, these discussions are raising more questions than answers, but such dialogue emphasizes the complexity that remains unaccounted for when using indicators like AHRs to evaluate health system performance or when making decisions regarding health care planning and service delivery.

A more comprehensive examination of population attributes vis-à-vis local health service systems is needed. Moreover, future analyses would benefit from the inclusion of additional variables (e.g., number of chronic conditions, health seeking behaviours and compliance with follow-up) to help interpret the results and provide a focus for future studies. Being unable to control for population factors such as education levels, disability levels, ethnicity and income, as well as age, gender and health region, limits our ability to index changes in the composition of the population and changes in service use over time.

Alternatively, it is also possible that regionalization is creating more vulnerable populations and subpopulations that are simply doing without care. As already discussed, there is a lack of data available to permit an assessment of levels of unmet need in the province. Indeed, this study has emphasized the critical importance of access to accurate, complete, and appropriate data to aid in understanding the multi-factorial relationships between need for care and use of services among older populations in BC. Ricketts et al. also underscore the need to use indicators like AHRs or ambulatory sensitive conditions, in conjunction with other information (e.g., physician supply) in order to evaluate health system performance [48].

Ultimately, it is also important to consider the choice of AHRs as a specific indicator of primary care system efficiency. While the literature indicates that this indicator reflects information about the availability of care, number of general practitioners, access to follow up care, practice patterns and political trends, AHRs have not been used extensively in Canadian contexts especially to compare care in both urban and rural areas [21,27,49]. Additionally, researchers caution that the indicator is not infallible since not all avoidable hospitalizations are likely to be 'avoided' under all circumstances [27]. With respect to the local service context, some research would suggest that inadequate follow-up and lack of patient compliance may be particularly salient issues relating to higher AHRs in rural communities [21].

This study has several limitations. For example, it must be cautioned that there are relatively few years of post-restructuring data to use in this assessment and thus comparisons can only be made across 11 years of data. In addition, the most substantial restructuring in the province occurred in the year 1997, yet, it could be that any changes associated with these shifts may only be observed over a longer period of time. Furthermore, changes that are apparent within the first few years immediately following restructuring may be the result of policy changes introduced at even earlier points in time. Finally, regarding the difficulty of making a clear link between regionalization and declining hospitalizations, changes may also reflect an initial, but temporary, period of transition associated with the re-definition of eligibility criteria, alterations of service levels and characteristics of target populations. Under these scenarios, it could be that service use may increase in future years. Thus, the length of time required for policy and structural changes in service delivery mechanisms to alter population-based patterns of service utilization requires further study and appreciation for how factors vary depending upon their scope and the implementation process. For this reason, generalizing from these findings to other locales remains tentative.

While not generalizable across provincial and international jurisdictions, primary care reform and health system restructuring and regionalization are prevalent trends across Canada and in other countries. In the struggle to reconcile issues of equity alongside issues of efficiency, it is hoped that this information will be relevant and useful for academics, planners, policy makers and decision makers. A key strength of this project has been the simultaneous consideration of both avoidable and non-avoidable hospitalizations across BC to help to interpret AHRs in a broader context of overall trends in hospitalization. Contemporary governments are concerned about cost-efficiency and related goals (for example, maximizing outcomes while minimizing the volume of service provided to a population and thereby minimizing costs [8,12,49]). But, the twin goals of efficiency and equity should not be de-coupled and considered in isolation from one another.

## Conclusion

These data suggest that access to effective primary care in rural communities remains problematic in BC. Additional research is needed to explore the discrepancy to determine the point at which social disparities in health become more extreme. For example, future analyses might want to recalculate AHRs and non-AHRs among different age groups, and by gender, to better understand trends in access to care over time. In addition, it would also be meaningful to take the rural and urban analysis to a more refined level, for example, looking at AHRs by specific local health planning areas in BC. In the last two decades, ongoing attention has focussed on the heterogeneity of 'places' (whether rural or urban) and their role in influencing health outcomes. Overall, the analyses employed here have underscored the importance of learning more about the broader needs and utilization patterns of older populations within specific local service contexts.

As our title suggests, a study such as this one is bedevilled by more questions than answers and by data limitations common in other health research. In addition, several waves of regionalization implemented across BC in the 1990s have served to complicate the picture rather than illuminate it. Observed declines in hospitalization across the spectrum from avoidable to non-avoidable hospitalizations have several competing explanations (e.g., changes in policy, program delivery, system capacity and/or access to human resources, service eligibility, and even changes in the health of older populations). Work remains to be done to address these issues and improve health care systems in terms of equity and efficiency. The need to provide high quality care to individuals in relation to their needs, regardless of where they live remains an important goal.

## Competing interests

The author(s) declare that they have no competing interests.

## Authors' contributions

DCF and MJP conceived and initiated this study using the BCLHD resource to investigate AHRs across BC as a measure of primary care system efficiency and to examine AHRs in relation to equity in access to care across urban and rural communities for older populations. DCF developed the first and second drafts of the manuscript. MJP provided content and critical feedback on each draft. ZC was responsible for the data analyses for the project and contributed to the methods write-up. EFD worked on the reference list and provided editorial feedback on drafts of the manuscript.

## Pre-publication history

The pre-publication history for this paper can be accessed here:



## Figures and Tables

**Table 1:** Avoidable Hospital Conditions and Corresponding ICD9-CM Codes

**Condition**	ICD-9-CM Codes*
Ruptured Appendix	540.0, 540.1
Asthma	493
Cellulitis	681, 682
Congestive heart failure	428, 402.01, 402.01, 402.11, 402.91
Diabetes	250.1, 250.2, 250.3, 251.0
Gangrene	758.4
Hypokalemia	276.8
Immunizable conditions	032, 033, 037, 072, 045, 055
Malignant hypertension	401.0, 402.0, 403.0, 404.0, 405.0, 437.2
Pneumonia	481, 482, 483, 485, 486
Pyelonephritis	590.0, 590.1, 590.8
Perforated or bleeding ulcer	531.0, 531.2, 531.4, 531.6, 532.0, 532.2, 531.4, 531.6, 532.0, 532.2, 532.4, 532.6, 533.0, 533.1, 533.2, 533.4, 533.5, 533.6

*ICD-9-CM (International Classification of Diseases, Ninth Revision, Clinical Modification).

Source: Weissman et al. [23]

**Table 2:** Comparison of Avoidable Hospitalizations Rates, non-Avoidable Hospitalization Rates and Total (First) Hospitalization Rates for BC for 1991–2000, Age 50+ population

**Year**	**AHR Standardized Rate/1000**	**URBAN AHR Standardized Rate/1000**	**RURAL AHR Standardized Rate/1000**	**AHRs relative rate (rural/urban)**
**1990**	10.2	9.7	14.4	1.49
**1991**	11.0	10.5	14.4	1.37
**1992**	10.6	10.1	14.1	1.40
**1993**	10.5	10.1	13.8	1.37
**1994**	10.2	9.8	13.1	1.34
**1995**	9.5	9.1	12.1	1.34
**1996**	9.9	9.4	12.7	1.34
**1997**	9.6	9.2	12.6	1.37
**1998**	9.3	8.9	12.1	1.37
**1999**	8.9	8.5	11.4	1.34
**2000**	7.8	7.4	10.7	1.45

**Year**	**Non-AHRS Standardized Rate/1000**	**URBAN non-AHR Standardized Rate/1000**	**RURAL non-AHR Standardized Rate/1000**	**Non-AHRs Relative rate (rural/urban)**

**1990**	186.8	183.2	212.1	1.16
**1991**	188.4	184.8	213.9	1.16
**1992**	184.0	180.2	210.2	1.17
**1993**	184.1	180.7	206.9	1.14
**1994**	177.8	174.5	200.1	1.15
**1995**	174.7	171.1	198.7	1.16
**1996**	172.4	168.5	199.0	1.18
**1997**	167.4	162.9	198.0	1.22
**1998**	162.6	158.4	190.9	1.21
**1999**	168.0	162.9	202.0	1.24
**2000**	164.8	159.9	198.1	1.24

**Year**	**Total Hospitalizations Standardized Rate/1000)**	**URBAN Total Hosps Standardized Rate/1000**	**RURAL Total Hosps Standardized Rate/1000**	**Tot-HRs Relative rate (rural/urban)**

**1990**	197.0	192.9	226.4	1.17
**1991**	199.4	195.3	228.3	1.17
**1992**	194.6	190.3	224.3	1.18
**1993**	194.7	190.8	220.8	1.16
**1994**	188.0	184.3	213.2	1.16
**1995**	184.2	180.2	210.9	1.17
**1996**	182.3	177.9	211.7	1.19
**1997**	177.0	172.0	210.5	1.22
**1998**	171.9	167.2	203.0	1.21
**1999**	176.9	171.5	213.4	1.24
**2000**	172.6	167.3	208.8	1.25

**Table 3:** Joinpoint Regression Results

**Analysis**	**Region**	**Joinpoint**	**Slope**	**Significance (p =)**
**AHRs**	Urban	JP = 0	-0.025	0.001
	Rural	JP = 0	-0.028	0.000
	Total	JP = 0	-0.026	0.000

**Non-AHRs**	Urban	JP = 0	-0.016	0.000
	Rural	JP = 0	-0.008	0.027
	Total	JP = 0	-0.015	0.000

**Total Hosps**	Urban	JP = 0	-0.017	0.000
	Rural	JP = 0	-0.010	0.001
	Total	JP = 0	-0.016	0.000

**Table 4:** T-test results for slope

**T-test Results**	**Significance and Decision**
*Comparing slopes within Avoidable Hospitalizations*	
Urban AHRs vs Rural AHRs	p > 0.05; do not reject Ho
*Comparing slopes within non-Avoidable Hospitalizations*	
Urban non-AHRs vs Rural non-AHRs	p < 0.005; reject Ho
*Comparing slopes within Total Hospitalizations*	
Urban tot-HRs vs Rural tot-HRs	p < 0.01; reject Ho
*Avoidable vs non-Avoidable Hospitalizations*	
Urban AHR vs Urban non-AHR	p < 0.01; reject Ho
Rural AHR vs Rural non-AHR	p < 0.01; reject Ho
Total AHRs vs Total non-AHRs	p > 0.05; do not reject Ho
